# Compact machine learning model for the accurate prediction of first 24-hour survival of mechanically ventilated patients

**DOI:** 10.3389/fmed.2024.1398565

**Published:** 2024-06-20

**Authors:** Quynh T. Nguyen, Mai P. Tran, Vishnu Prabhakaran, Andrew Liu, Ghi H. Nguyen

**Affiliations:** ^1^Department of Mathematics and Statistics, Langara College, Vancouver, BC, Canada; ^2^Emergency Department, 108 Military Central Hospital, Hanoi, Vietnam

**Keywords:** machine learning models, Autofeat, tree-based algorithms, non-tree based algorithms, mechanically ventilated patients, dimension reductions

## Abstract

**Background:**

The field of machine learning has been evolving and applied in medical applications. We utilised a public dataset, MIMIC-III, to develop compact models that can accurately predict the outcome of mechanically ventilated patients in the first 24 h of first-time hospital admission.

**Methods:**

67 predictive features, grouped into 6 categories, were selected for the classification and prediction task. 4 tree-based algorithms (Decision Tree, Bagging, eXtreme Gradient Boosting and Random Forest), and 5 non-tree-based algorithms (Logistic Regression, K-Nearest Neighbour, Linear Discriminant Analysis, Support Vector Machine and Naïve Bayes), were employed to predict the outcome of 18,883 mechanically ventilated patients. 5 scenarios were crafted to mirror the target population as per existing literature. S1.1 reflected an imbalanced situation, with significantly fewer mortality cases than survival ones, and both the training and test sets played similar target class distributions. S1.2 and S2.2 featured balanced classes; however, instances from the majority class were removed from the test set and/or the training set. S1.3 and S 2.3 generated additional instances of the minority class via the Synthetic Minority Over-sampling Technique. Standard evaluation metrics were used to determine the best-performing models for each scenario. With the best performers, Autofeat, an automated feature engineering library, was used to eliminate less important features per scenario.

**Results:**

Tree-based models generally outperformed the non-tree-based ones. Moreover, XGB consistently yielded the highest AUC score (between 0.91 and 0.97), while exhibiting relatively high Sensitivity (between 0.58 and 0.88) on 4 scenarios (1.2, 2.2, 1.3, and 2.3). After reducing a significant number of predictors, the selected calibrated ML models were still able to achieve similar AUC and MCC scores across those scenarios. The calibration curves of the XGB and BG models, both prior to and post dimension reduction in Scenario 2.2, showed better alignment to the perfect calibration line than curves produced from other algorithms.

**Conclusion:**

This study demonstrated that dimension-reduced models can perform well and are able to retain the important features for the classification tasks. Deploying a compact machine learning model into production helps reduce costs in terms of computational resources and monitoring changes in input data over time.

## 1 Introduction

Accurate survival prediction for mechanically ventilated patients in intensive care units (ICUs) remain a medically challenging feat (1, 2). To this end, various studies attempted to leverage complex machine learning (ML) algorithms trained on large databases combining clinical facts and involving thousands of patients. One example is the classification paradigms where supervised ML algorithms were used in developing predictive models, such as Decision Tree (DT), Bagging (BG), or eXtreme Gradient Boosting (XGB). Ruan et al. (3) analysed 162,200 episodes of respiratory failure, included in the Taiwanese National Health Insurance database, to study how the prognosis of mechanically ventilated patients changed with each additional day required for treatment. Another study conducted by Li et al. (4) utilised 4,530 patients’ records, along with medical facts, to predict hospital mortality in patients with congestive heart failure, and where mechanical ventilation was deployed, by leveraging 11 ML algorithms.

The issue of providing accurate prognostic estimations is also important for both patients and their decision-makers since the number of patients requiring prolonged mechanical ventilation has increased during the last decade (5). This has also been particularly underscored as a result of the Covid-19 pandemic. By employing various ML algorithms to predict the outcomes of mechanically ventilated patients, clinicians can expedite the decision-making process (6). Zhu et al. (7) used seven ML methods on 25,659 ICU adults to estimate the survival of mechanically ventilated patients. The highest Area Under the Curve (AUC) score attained from the testing set was 0.821 for the XGB classifier, and the calibration curve was closely aligned with the perfect predicted probability line. In the study, the dataset used for the training and testing tasks was balanced, with the non-surviving group of patients accounting for 45.5%. However, it should be noted that there is great location-dependent variability pertaining to mortality. For example, the mortality rate of ICU patients who needed mechanical ventilation in Brazil was over 50% (8), while in Saudi Arabia the adult mortality rate of mechanically ventilated patients was 29% (9). Therefore, our study aimed at conducting its analyses using various scenarios drawn out of the real world, including balanced and imbalanced class distributions that reflect a target population.

Searching a large space of hyper-parameters can be time-consuming and computationally costly, hindering the optimisation process. For example, the time complexity computed for an unpruned decision tree is $O\left(p.\mathrm{nlog}\left(n\right)\right)$ , where p is the number of features and n is the number of observations (10). Zhu et al. (7) used all available predictors and observations for the prediction tasks. This means the computational time during the training phase could be significant since the number of node evaluations could be in the range of millions. While Zhu et al. (7) used the Random Forest (RF) algorithm in their study, Louppe (11) and Do et al. (12) noted that the efficiency and performance of RF models may decrease as the number of features increases. This presents visible limitations when high-dimensional data and numerous observations are required for re-training ML models during the deployment and monitoring of ML applications, including (1) timely delivery of results while ingesting various data sources into ML models, (2) cost to monitor changes in input data, as it can deviate from the training dataset over time, so called data drift, and for quality control of model updates (13). Hence, this study proposes a method to automate feature selection tasks, aiming to reduce the number of predictors while endeavouring to achieve a better, or at least similar, classification accuracy (AUC score), compared to the one reported by Zhu et al. (7).

In the context of this study, a set of assumptions was outlined:

- Three main test sets were employed to reflect different real-world scenarios: 1 test set with imbalanced classes, and 2 test sets with balanced classes achieved through under-sampling to remove instances of the majority (survival) class and SMOTe over-sampling to increase instances of the minority (mortality) class.
- The mortality class was a minority in the final dataset that we extracted from MIMIC-III. Therefore, it was considered as a positive class when we evaluated various metrics in relation to the ML models’ performance. Conversely, the survival class was the majority, or negative, class or value.
- True positive or true negative is categorised if a mechanically ventilated patient was predicted correctly as dead or alive, respectively. False positive and false negative occurred in misdiagnosed cases.
- We assumed in this study that the consequences of overlooking mortality cases far outweigh the harms of unnecessary treatment; the prognosis for ML models with a higher Sensitivity or Recall were favoured.

## 2 Materials and methods

### 2.1 Data source

Data for this study was collected from the Medical Information Mart for Intensive Care III (MIMIC-III) database, which is publicly available. After de-identifying their records, it consisted of 46,520 patients who stayed in the critical care unit of Boston’s Beth Israel Deaconess Medical Centre between 2001 and 2012. The predictive models of this study were trained and validated using the retrospectively extracted data therein. It should also be noted that this study was performed based on the analysis of Zhu et al.’s study (7) and Johnson et al.’s report (14).

### 2.2 Inclusion criteria of study population

In this paper, the study population along with its target variable and predictors were extracted from the MIMIC-III database using a Structured Language Query (SQL) script. The data selection criteria follow the process presented by Zhu et al. (7). There were 6 distinct groups of predictors, namely, demographic characteristics, medical history, disease severity, diagnosis, vital signs and laboratory results. The criteria for each group are described below:

- Demographic characteristics: the subject IDs and ICU admission times were used to identify distinct adult patients and their first ICU admission. Initially, we extracted patients who were 16 years or older. Age was calculated using the difference between the date of birth and the date of hospital admission.
- ICD-9 code provided by MIMIC-III was used to define the medical history features. The rationale of using ICD-9, and not ICD-11 is due to the design of the MIMIC-III.
- In the disease severity group features, we extracted the SAP II, SOFA and OASIS scores.
- In the diagnostic features group, diagnosis of sepsis, any organ failure and/or organ dysfunction were used to select the population. Also, diagnosis of severe clinical issues, such as respiratory, coagulation, liver, cardiovascular, central nervous system or renal failure, were identified if the SOFA score of the respective organ or system reached 4.
- In the vital signs and laboratory results groups, only values taken in the first 24 h of a patients’ presentation were taken into consideration.
- For the ventilation duration group, we used the official scripts provided by MIMIC-III for data filtering.

Once all the criteria were established, filtering was applied to limit the patient pool to those within 18–90 years old who were on mechanical ventilation. As a result, we eliminated 2,074 ICU patients who were outside the prescribed age range, and we also excluded 17,635 patients who were not subjected to mechanical ventilation. Several other irrelevant features were dropped, which ultimately resulted in a dataset consisting of 18,883 patients and 67 predictors. The target variable for the predictive modelling process was hospital mortality.

### 2.3 Data exploration and pre-processing

Missing values, outliers and valid value ranges were processed prior to getting a final dataset. Outliers were not removed since they might carry important information. At the same time, inclusion of outliers might affect the robustness of the models. To lessen possible impacts, the data was validated against acceptable ranges for all numerical variables (vital signs and laboratory results) to ensure that exceptionally egregious values were excluded from the modelling process. For example, outlier detection was conducted using a range of 3 standard deviations, preserving the integrity of the data and its representation of real-world measurements. The valid ranges of all features are provided, along with the feature descriptions, in Supplementary material S1.

Median imputation by outcome class was employed to handle missing values, including Max Lactate, Min Lactate and Mean Lactate since each has about 27% missing in this study. This imputation aimed to minimise bias resulting from outliers and to maintain uniformity within the classes. Moreover, Zhu et al.’s approach (7), which set the threshold for missing value removal at 30%, solidified the decision to impute missing values rather than remove any features.

Inconsistencies were observed in the ICD-9 codes used for broader medical terms such as malignancy and stroke across multiple publications, posing challenges for reliable disease classification. To ensure consistency and reproducibility, the same codes used in the publication by Feng et al. (15) for malignancy and stroke were adopted.

### 2.4 Data sampling

**Figure 1:**
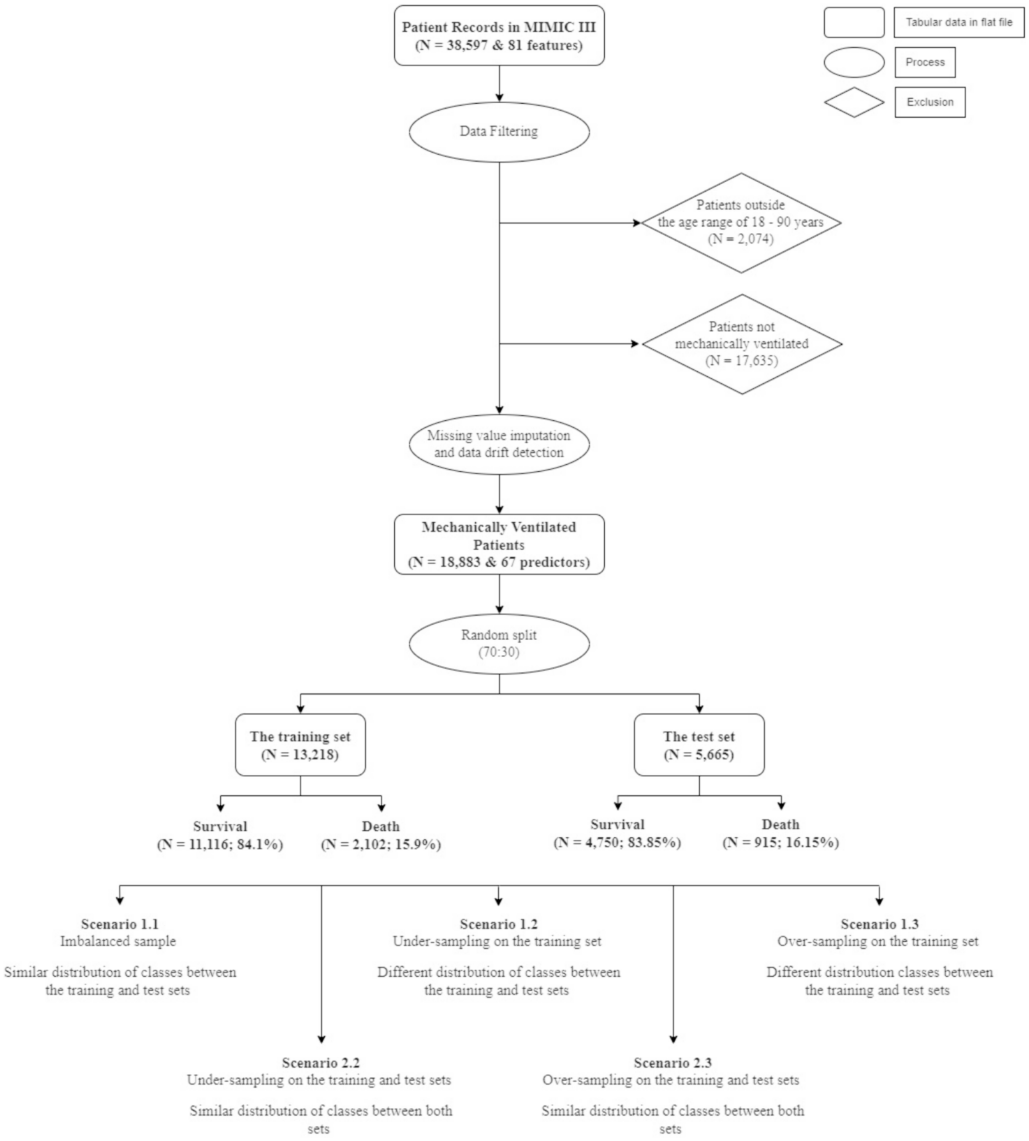
Data selection and scenarios creation.

The total size of the final dataset for analyses included 18,883 patients and a set of 67 features. Although the case of classes, where the number of deaths is considered as a minority, this was not considered in Zhu et al.’s study (7). To deal with this issue, as well as the possibility of data drift when deploying the ML models, we created 5 different scenarios to compare the results of the predictive models using random under-sampling and Synthetic Minority Over-sampling Technique (SMOTe). First, we randomly split the data (*N* = 18,883) using a ratio of 70:30. The rationale of using this train-test split is based on the study conducted by Singh et al. (16) who reported that using 70:30 ratio gives the highest AUC scores among different split ratios, across various ML models, including Logistic Regression (LR), Linear Discriminant Analysis (LDA), Random Forest (RF), and Naïve Bayes (NB). Using this initial split also ensured that all instances in the test set were not presented to all ML models while training. Next, we applied random sampling and SMOTe to either under- or over-sample the training and the test sets separately (Figure 1).

The first situation, including scenario 1.1 (S1.1), 1.2 (S1.2), and 1.3 (S1.3), aimed at keeping the test set (*N* = 5,665) unaltered, while different sampling techniques were applied on the training set to achieve better learning for the minority class. This created a situation where the size of the test set was larger than the training set (S1.2). The sampling techniques adopted in this study comprised random under-sampling and SMOTe. The second situation, which comprised scenario 2.2 (S2.2) and 2.3 (S2.3) used the same sampling techniques to create balanced classes for both the training and test sets. Table 1 gives the details of the sizes of different samples.

**Table 1:** Binary class distribution per scenario.

Scenario	Class	Training sets (%)	Testing sets (%)
S1.1	Survival	11,116 (84.1%)	4,750 (83.85%)
	Mortality	2,102 (15.9%)	915 (16.15%)
Total	18,883	13,218	5,665
S1.2	Survival	2,102 (50%)	4,750 (83.85%)
	Mortality	2,102 (50%)	915 (16.15%)
Total	9,869	4,204	5,665
S1.3	Survival	11,116 (50%)	4,750 (83.85%)
	Mortality	11,116 (50%)	915 (16.15%)
Total	27,897	22,232	5,665
S2.2	Survival	2,102 (50%)	915 (50%)
	Mortality	2,102 (50%)	915 (50%)
Total	6,034	4,204	1,830
S2.3	Survival	11,116 (50%)	4,750 (50%)
	Mortality	11,116 (50%)	4,750 (50%)
Total	31,732	22,232	9,500

### 2.5 Dimension reduction Autofeat tool

The second objective of this study was to explore the possibility of automating the feature engineering process by reducing the 67 independent variables to a significantly smaller number of features, while achieving a similar performance.

We identified 2 existing packages on automating features, namely, Deep Feature Synthesis (DFS) and Autofeat. First, an experiment was conducted using DFS, which was to automatically generate new features based on relationships between the variables in a dataset. However, many manual steps were still required, such as manually selecting aggregative/transformative features on finding optimal combination of primitives. Note that in this package, feature primitives are refined as building blocks for creating features. Moreover, another limitation of DFS was that the package conducts feature selections within the memory of one machine. This was deemed to be very time consuming, and therefore, was decided not to use DFS for this objective.

We employed the Autofeat Python package to automatically reduce features. The advantage of using Autofeat is the interpretability after reducing dimensions. The Autofeat package is an automated feature engineering and selection tool developed for use in scientific use cases where measurements are stored in a single table (17). It provides the AutofeatRegressor and AutofeatClassifier classes, which automatically generate non-linear forms of the input variables in the original data prior to selecting the most relevant features and fitting them to a linear prediction model. A third class, called FeatureSelector, is also present as part of the library which provides only the feature generation and selection part without the modelling step. The FeatureSelector class works by first generating a non-linear set of features from the original set of features by alternating multiple times between steps of applying non-linear transformation like log, square root, inverse, square, cube, trigonometric transformations, etc., to the variables and combining pairs of these features through calculating their sum, difference and product. This leads to an exponentially growing feature space, with, for example, 3 original features growing to 4,000 features by the third step. In addition to this, the Pint Python library was also used to generate dimensionless features from the original ones by applying the Buckingham π-theorem. This ensured that only valid features were generated, and no feature – for example the difference between a feature depicting a time measure and another depicting temperature – was generated. Memory management in the non-linear feature generation step can be accomplished by initially sub-sampling the data points.

The goal was to only select these features from the thousands generated that made a meaningful contribution when added as input to a linear model. To achieve this, firstly features that were highly correlated to the original features, or with simpler features, were removed prior to using a multi-step selection process on the remaining features. Instead of dropping features that did not contribute any information by themselves, or might seem redundant, a Lasso LARS regression model and an L1-regularised LR model were used for noise filtering in which a model was trained on the original features as well as noise features generated by shuffling data or random sampling from a normal distribution. Only features with model coefficients greater than the largest coefficient of the noise features were kept. To avoid the problem faced when modelling using a large, interrelated feature space on a smaller number of data points, an L1-regularised model was first trained on all features, and those with the largest absolute coefficients were selected. The remaining features were then divided into equal chunks of size smaller than half of the total number of features, and a model was fitted to each chunk to determine which features to add. The subset of features thus obtained was merged before fitting a final model to determine the final set of features. This selection process was performed multiple times with different sub-samples of the data to obtain a more robust feature set. The feature results of these runs were merged before highly correlated features were filtered out again and a model was fitted on the remaining set to identify the final selected predictors. The number of features for different sample methods are shown in Table 2.

**Table 2:** A breakdown of the total number of predictors post dimension reduction.

Scenario	Total number of predictors	
	Prior to Autofeat	Post Autofeat
S1.1 – Imbalanced	67	29
S1.2 and 2.2 – Under-sampled	67	22
S1.3 and 2.3 – Over-sampled	67	40

### 2.6 Machine learning models for predictive modelling

In this study, models were developed to predict mortality in the first 24 h of ICU admission for mechanically ventilated patients using 4 different tree-based techniques including DT, BG, XGB and RF, along with 5 other non-tree-based algorithms – LR, K-Nearest Neighbour (KNN), LDA, Support Vector Machine (SVM) and NB.

To enhance the model performance, both the grid search strategy, which considers all viable combinations of hyper-parameters, and its implementation in Scikit-learn, namely GridSearchCV, were used to identify the optimum parameter values. The parameters to tune were “estimator,” “param grid” and “cv.” The main objective for this tuning process was to improve the Recall score. Some of the tree-based algorithms, such as XGB, or non-tree based, namely, SVM, use resources extensively. Hence, we only limited 5 K-fold cross-validation.

Additionally, all the selected ML models in this study were also fed into calibrated classifiers to improve the reliability of their predicted probabilities, and subsequently utilised the grid search strategies to identify the best parameters.

### 2.7 Model assessment

In terms of discrimination capability, the confusion matrix metrics of accuracy, including Precision, Recall and F1-score, and the AUC score were used to assess the best performance models. Based on the prediction probabilities, the Receiver Operating Characteristic (ROC) curves were developed. Then, the model with the best predictive performance was identified by comparing the AUC values of the models in the testing sets, while F1-score, Precision and Recall were used to understand the behaviours of the ML models towards the discrimination ability.

Log Loss and Brier Score were employed to select the best models that have the highest accurate prediction probabilities. Brier Score comprises 2 elements of measures, calibration and discrimination. The term “discrimination” in the Brier Score decomposition is more about the spread or variability of predicted probabilities across instances of different classes, whereas ROC curves entail how well a model separates two classes, regardless of the threshold value. In other words, AUC explicitly focuses on the ability of a model to discriminate between positive and negative classes based on rank ordering, while discrimination of the Brier Score indicates the variability of predicted probabilities across instances of different classes. In addition, the calibrated curve, or reliability graph, was used to evaluate how well calibrated a model could be. According to Assel et al. (18), “a well-calibrated model has a better Brier score than a miscalibrated model with similar discrimination.” However, “the Brier score does not perform well in several common situations. Specially, the Brier score will favour a test with high specificity where the clinical context requires high Sensitivity if it is the case that prevalence is low.” Hence, our study would use the Brier Score to only make overall observations.

ML algorithms generally expect balanced class distributions with equal cost for classification problems. As such, these algorithms are not efficient in handling complicated imbalanced data sets (19). In addition, ML algorithms contribute more towards performing higher quality classification of the majority class samples, which are assumed as more significant. As such, learning algorithms exhibit bias towards classes containing more samples. However, in the medical field, cases of imbalanced class distribution are predominant. This may create a situation where a ML classifier might perform worse in production systems than in the development environment. Hence, it is important to identify a ML algorithm that has a stable performance in both situations, imbalanced and balanced class distribution. In this study, we assumed that the imbalanced test set with the prevalence of an event (mortality) of nearly 20% was similar to the future data in production environments. In this case, S1.1, S1.2 and S1.3 used such imbalanced test set. S2.2 and S2.3 were simulated to reflect a real-life situation where the mortality and survival groups had similar distributions (approximately 50%). In details, S2.2 randomly removed instances from the majority class (to survival class) while S2.3 used SMOTe to increase instances from the minority class (mortality group).

All models were calibrated, and the results presented in this study are based on the output of the best parameters using GridSearchCV of calibrated models. Moreover, we present Matthew Correlation Coefficients (MCC) in the situation where we endeavour to see the changes in prediction performance prior and post dimension reduction. Sensitivity is one of the metrics to be used to select the best performer when models have similar AUC scores.

### 2.8 Statistical analysis

The statistical characteristics of the survival and mortality cohorts were compared. The comparison results are demonstrated in Supplementary material S1. Binary features were expressed in count and percentage, while numerical features were expressed in median and interquartile range (IQR). Except for Uncomplicated Hypertension, Uncomplicated Diabetes, Complicated Diabetes, Peripheral Vascular Disease and Hypothyroidism, the mortality cohort tended to have a higher count, or median value, than the survival cohort. There were also more male patients in the survival cohort. Features that had a large difference in percentage or IQR (> 10) between the 2 groups were: Hematologic Disease, Liver Disease, Sepsis, Any Organ Failure, Severe Cardiovascular Failure, Severe Renal Failure, Respiratory Dysfunction, Cardiovascular Dysfunction, Renal Dysfunction, Hematologic Dysfunction, Metabolic Dysfunction, Max Glucose, Min Glucose, Mean Glucose, Max BUN, Min BUN and Mean BUN.

The D’Agostino K-squared test, recognised for effectively assessing data distribution characteristics as demonstrated by Yoshida et al. (20), was utilised to identify numerical features that did not follow a normal distribution, and the results are included in Supplementary material S2. To ensure that the use of median imputation to fill missing values does not change the original distribution of the 67 features, Kullback–Leibler (KL) divergence, which is a measure (in “nats”) of the disparity between a probability distribution and a reference probability distribution, was used. Following the procedure set by Cover and Thomas (21) for KL divergence test, it was observed that the KL divergence results for all 67 variables were very low. All binary and Age variables had a divergence of 0 nats; among all the numeric variables, Max Lactate, Mean Lactate and Min Lactate had the top three divergences of 0.052 nats, 0.050 nats and 0.049 nats, respectively. This was expected due to the large percentage of missing values in these variables. Nonetheless, these values were deemed to be very low and indicated that median imputation did not adversely affect the distributions of the features.

**Figure 2:**
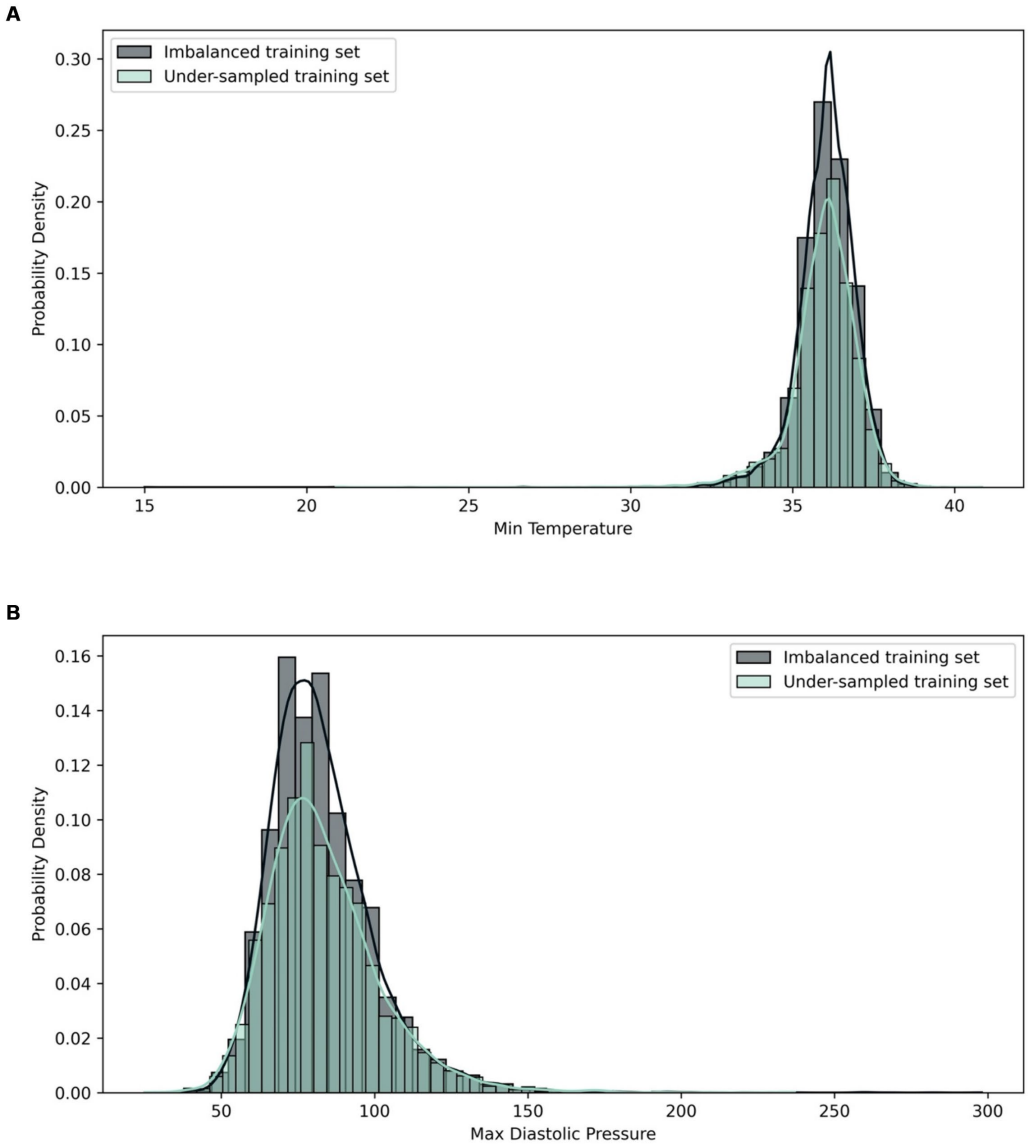
Histogram comparing distribution of **(A)** Min Temperature and **(B)** Max Diastolic Pressure in imbalanced and under-sampled training set.

KL divergence was also utilised to compare the distribution pre- and post-application of a sampling method on the training sets. The divergence score was calculated for each feature and compared between the imbalanced training set and sampled training sets to study the effects of sampling on the feature distributions. It was observed that in the case of over-sampled data, all features had very low KL divergence metrics, with SAPS II having the highest score of 0.04 nats. In the case of under-sampled data, all variables had very low divergence metrics, except for Min Temperature with 0.76 nats and Max Diastolic Pressure with 0.27 nats. The high KL divergence score might be attributed to the difference in the minimum and maximum values related to the original and post-sampling distributions. A closer examination in the histograms shows that the distributions are very similar in terms of shapes (Figure 2). The results of comparison are attached in Supplementary material S3.

## 3 Results

This study aims to develop a compact-predictive model that can discriminate binary classes for mechanically ventilated patients and generate accurate predicted probability of outcomes. The approach that we took was first to identify the ML models that best perform across different scenarios, using all 67 features. Once the best performers were established, Autofeat was utilised to select the most important features for all scenarios (Table 2). We then constructed and trained those identified models on the selected features and tested their respective performances.

### 3.1 Prior to dimension reductions

#### 3.1.1 Scenario-based analysis

Since we aim to maximise the correct prediction on the minority class, models that have high values of Sensitivity would be considered among other metrics. In cases of balanced classes, Macro Average is the same as weight average, and for imbalanced classes, we assessed performance using Weighted Average of Recall, Precision and F1-score. Hence in this sub-section we used Weighted Average metrics to access the performance of those models.

##### 3.1.1.1 Scenario 1.1 – Imbalanced class | similar class distribution between the training and test sets | no sampling technique

RF, BG and XGB were the only 2 out of 3 models that have a Sensitivity higher than 50%. Other models yielded Sensitivity below 50%. RF has the highest Sensitivity (54%) and the second highest of AUC (92%), with relatively low Log Loss and low Brier score. Additionally, the highest Weighted Average Recall and F1-score values of 0.9 and 0.89, respectively, resulted from RF. In terms of accurate predicted probability, the calibration curve entailed that the RF model was the most closely aligned with the perfect line. However, the curve showed that this model was consistently under-predicting the mortality group.

##### 3.1.1.2 Scenario 1.2 – Imbalanced class | dissimilar class distribution between the training and test sets | under-sampling method on the training set

While the XGB and BG models produced the highest scores of AUC, Weighted Average Recall and F1-score, these models exhibited a 5% lower Sensitivity score than those produced by both the RF and LR models. In terms of Log Loss and Brier Score values, the XGB and BG models had values below the mean values, while the RF and LR scores were higher than the means. The calibration curves showed that RF and LR were consistently and severely over-predicted the mortality group, while the XGB and BG models did not. This finding was reflected by the means of calibration curves. While the Sensitivity margin difference between the XGB/BG and RF/LR models was approximately 5%, the gap between Weight Average Recall and F1-score was 20%, hence, XGB and BG would be performed more accurately in this scenario.

##### 3.1.1.3 Scenario 1.3 – Imbalanced class | dissimilar class distribution between the training and test sets | over-sampling method on the training set

XGB had the highest weighted Recall, F1-scores, and AUC value among all algorithms. Also, Brier score and Log Loss of XGB were way below the mean values. However, the Sensitivity score was at the low end (0.58). LR had the highest Sensitivity score and relatively high AUC (0.827), but the Log Loss was much higher than the Log Loss of XGB. The calibration curve showed that LR highly overestimated while XGB underestimated the mortality group. Therefore, both models were selected for this scenario.

##### 3.1.1.4 Scenario 2.2 – Balanced class | similar class distribution between the training and test sets | under-sampling method on the training and test sets

BG and XGB had relatively high Sensitivity scores, 0.890 and 0.884, respectively, and the highest respective AUC of 0.921 and 0.928. The Brier scores for both models were below the mean and median. The same applied to Log Loss. Weighted Average Recall and F1-score of these 2 algorithms were the highest. The calibration curve projected that both models slightly overestimated the mortality group.

##### 3.1.1.5 Scenario 2.3 – Balanced class | similar class distribution between the training and test sets and over-sampling method on the training and test sets

XGB had the highest AUC, Weighted Recall and F1-score. Log Loss and Brier score were below the mean while the Sensitivity was relatively high.

Table 3 shows all evaluation metrics of the best models per scenario. These algorithms will eventually be selected in Table 4 to perform dimension reduction.

**Table 3:** Best models per scenario.

Scenario	Model	Precision	Recall	F1-score	Sensitivity	Specificity	AUC	Brier score	Log Loss
S1.1	RF	0.894	0.900	0.893	0.540	0.970	0.922	0.020	0.105
S1.2	BG	0.889	0.806	0.828	0.890	0.790	0.920	0.053	0.200
S1.2	XGB	0.891	0.817	0.837	0.884	0.805	0.926	0.050	0.193
S2.2	BG	0.847	0.844	0.844	0.884	0.803	0.927	0.053	0.205
S2.2	XGB	0.846	0.844	0.843	0.861	0.801	0.914	0.058	0.223
S2.3	XGB	0.899	0.896	0.895	0.851	0.941	0.969	0.017	0.092
S1.3	XGB	0.878	0.883	0.880	0.583	0.941	0.905	0.017	0.093
S1.3	LR	0.853	0.613	0.662	0.889	0.560	0.827	0.075	0.290

**Table 4:** MCC score prior to and post Autofeat.

Scenario	Model	MCC		AUC [95% CI]	
		Prior to Autofeat	Post Autofeat	Prior to Autofeat	Post Autofeat
S1.1	RF	0.561	0.536**↓**	0.92 [0.91–0.93]	0.91 [0.90–0.92]
S1.2	BG	0.537	0.507**↓**	0.92 [0.91–0.93]	0.91 [0.90–0.92]
S1.2	XGB	0.550	0.506**↓**	0.93 [0.92–0.93]	0.91 [0.90–0.92]
S1.3	XGB	0.548	0.556**↑**	0.90 [0.90–0.91]	0.90 [0.89–0.91]
S1.3	LR	0.409	0.429**↑**	0.84 [0.83–0.86]	0.85 [0.84–0.86]
S2.2	BG	0.691	0.658**↓**	0.92 [0.91–0.93]	0.91 [0.90–0.92]
S2.2	XGB	0.690	0.638**↓**	0.93 [0.92–0.93]	0.91 [0.90–0.91]
S2.3	XGB	0.794	0.795**↑**	0.97 [0.97–0.97]	0.97 [0.96–0.97]

#### 3.1.2 Analysis of balanced classes in test sets and similar distribution between the training and test set

**Figure 3:**
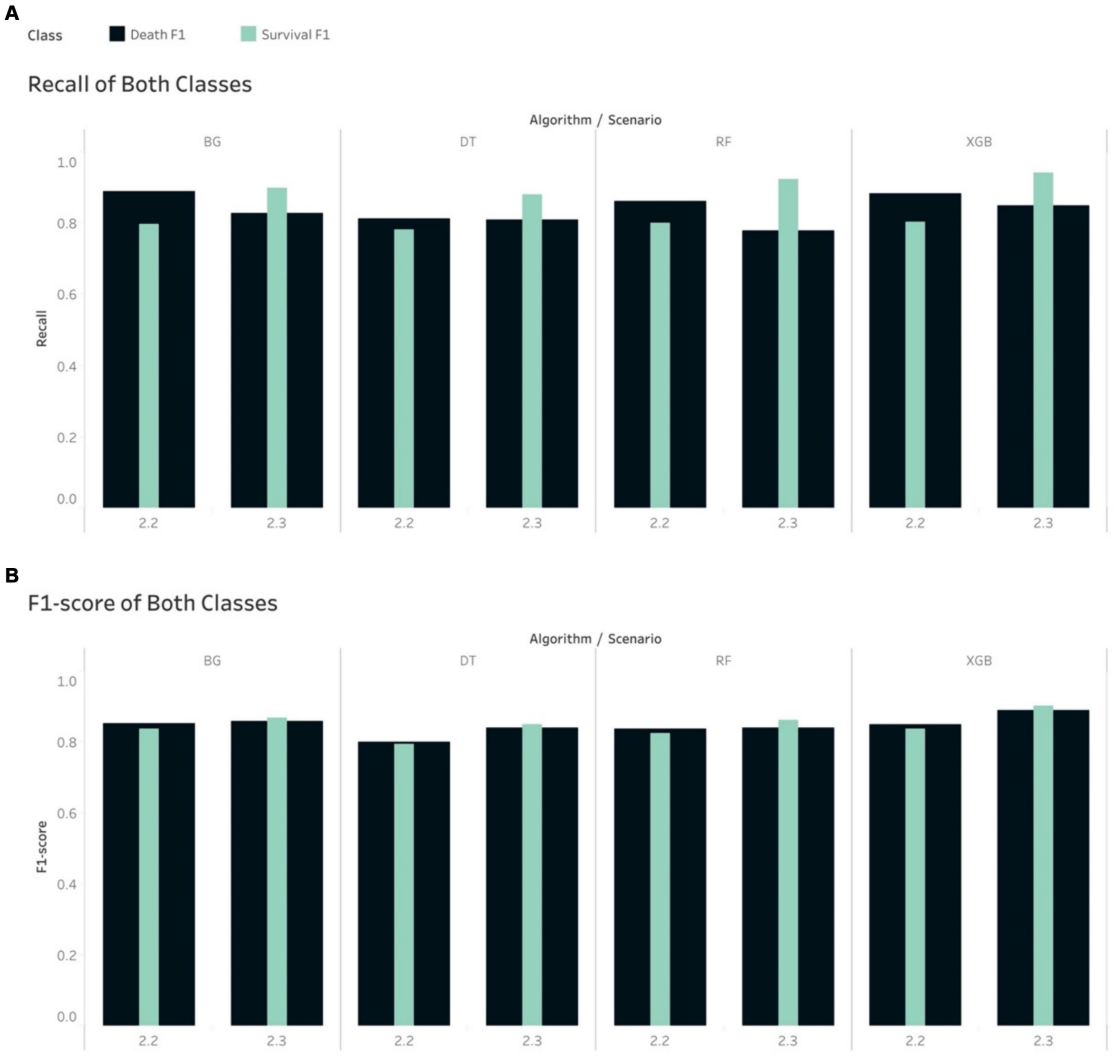
Comparison of calibrated tree-based models in scenario 2.2 and 2.3 using **(A)** Recall of both classes and **(B)** F1-score of both classes.

The Recall scores for the mortality class were higher than their counterpart (survival group) in relation to S2.2, where the majority class (survival group) was under-sampled across all 4 tree-based algorithms. The opposite trend was seen in S2.3, where the minority class was over-sampled to be balanced (Figure 3). This had some impacts on the F1-score, where the tree-based algorithms gave higher scores for the mortality class, in S2.2, than the survival group. The opposite trend was found in S2.3, where F1-score for the survival groups were slightly higher than the mortality group (Figure 3).

The non-tree-based algorithms yielded a mixture of Recall and F1-score for both classes in both S2.2 and S2.3. For example, the Recall and F1-score for the mortality class were higher than their counterpart, except for NB if looking at S2.2. However, with regards to S2.3, the 2 non-tree-based models depicted a slightly different pattern than tree-based models (higher Recall scores for the mortality group), except KNN, LDA and NB. This led to a mixture of F1-score for both groups and for all models in S2.3.

To achieve higher level of interpretation, the Weighted Average of Precision, Recall and F1-score were considered. Table 5 shows that all tree-based models gave higher Weighted Average Precision, Recall and F1-score when performing on S2.3 compared to S2.2. Among the tree-based models, DT generated the lowest weighted Precision (0.799), Recall (0.798) and F1-score (0.798) score with regards to S2.2. Among the non-tree-based models, LR resulted in the highest weighted Recall score (0.789) and F1-score (0.788) with regards to S2.2. The same pattern could be depicted for S2.3. This finding could lead to an initial conclusion that that the tree-based models, overall, performed better that the non-tree-based models in terms of the classification and prediction the binary outcome (class discrimination) when the target population had a balanced distribution class situation.

**Table 5:** Weighted average of precision, recall and F1-score for all algorithms.

Scenario	Model	Precision	Recall	F1-score
S2.2	DT	0.80	0.80	0.80
S2.3	DT	0.85	0.85	0.85
S2.2	BG	0.85	0.84	0.84
S2.3	BG	0.87	0.86	0.86
S2.2	XGB	0.85	0.84	0.84
S2.3	XGB	0.90	0.90	0.90
S2.2	RF	0.83	0.83	0.83
S2.3	RF	0.86	0.85	0.85
S2.2	KNN	0.87	0.70	0.74
S2.3	KNN	0.83	0.71	0.74
S2.2	LDA	0.78	0.78	0.78
S2.3	LDA	0.84	0.84	0.84
S2.2	SVM	0.76	0.55	0.44
S2.3	SVM	0.79	0.70	0.68
S2.2	NB	0.74	0.72	0.72
S2.3	NB	0.73	0.72	0.72
S2.2	LR	0.79	0.79	0.79
S2.3	LR	0.82	0.82	0.82

#### 3.1.3 Analysis of imbalanced classes and dissimilar distribution between the training and test set

**Figure 4:**
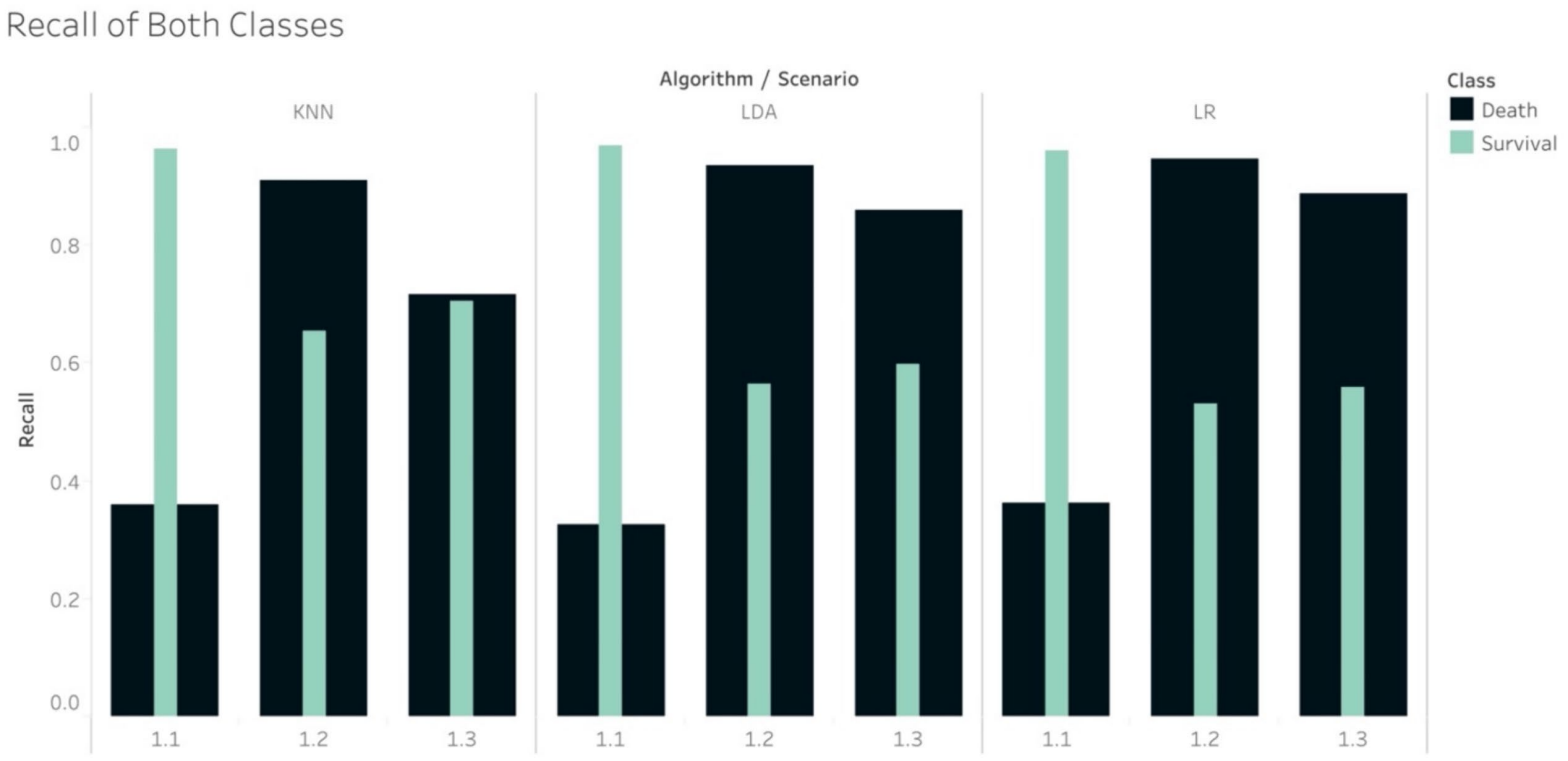
Recall of non-tree-based models in scenario 1.1, 1.2 and 1.3.

The Recall scores produced by the tree-based models for the mortality class on in S1.2 were higher than those for the survival group. Note that in this scenario, the random under sampling method was utilised on the training set, while the test set remained imbalanced. This finding is similar to the pattern that was found in S2.2. In S1.3, the Recall scores for the survival group were higher than those for the mortality group for all the tree-based algorithms, except RF. This finding was, again, similar to that of S2.3. With regard to S1.3, if using the non-tree based algorithms, namely LR, KNN and LDA, the Recall scores were higher for the mortality group, although the size of the mortality group was increased using SMOTe to match the size of the survival group during the training process (Figure 4). The case of S1.1, where there were imbalanced classes in both training and test sets, the Recall and F1-score of the survival group were much higher.

**Figure 5:**
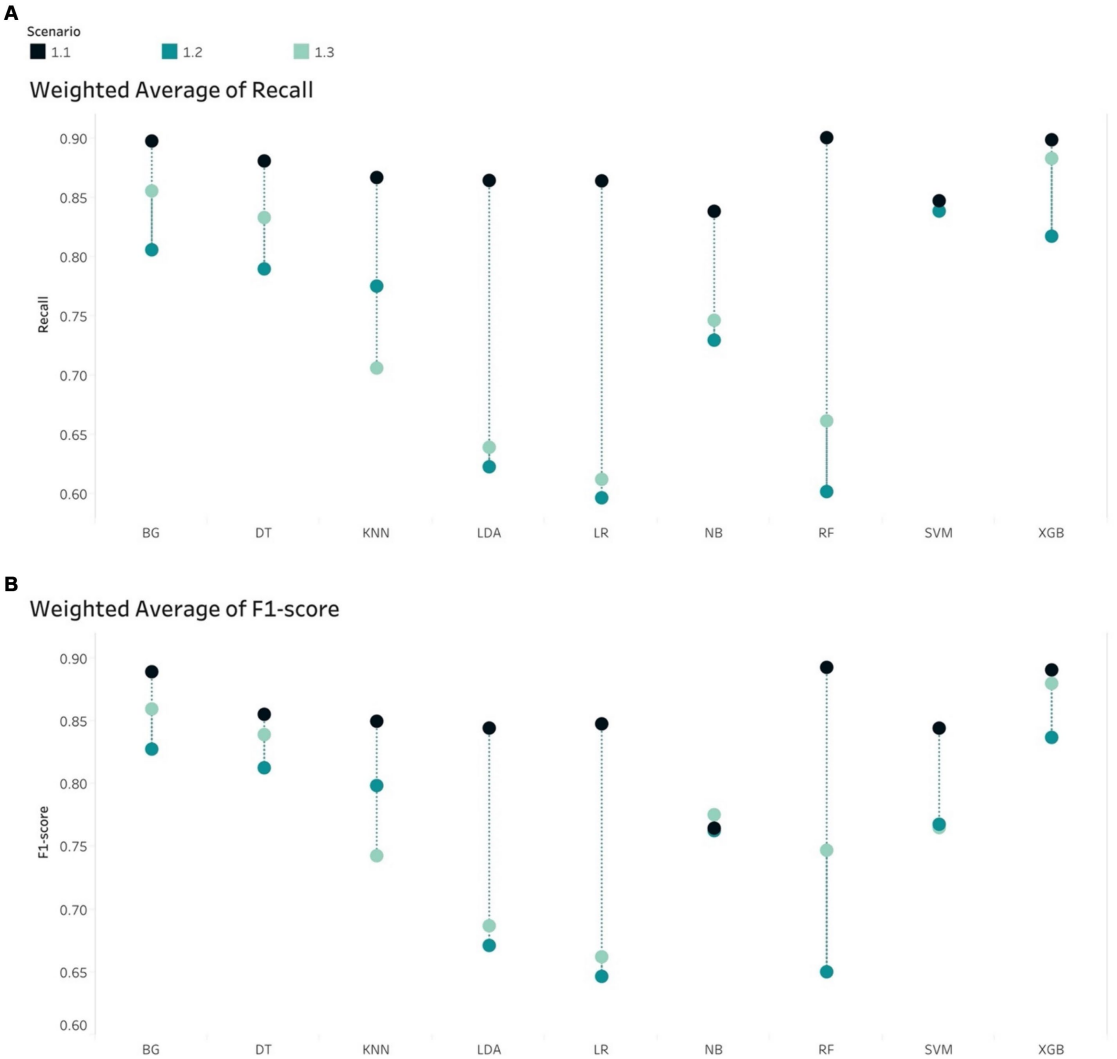
Weight Average of **(A)** Recall and **(B)** F1-score, all algorithms in the imbalanced scenarios.

In the case of imbalance in the test set, all classifiers resulted in the best values of Weighted Average Recall and F1-score in S1.1, except NB (Figure 5). F1-score confirmed that the tree-based classifiers outperformed the non-tree-based.

#### 3.1.4 Analysis of AUC, Log Loss and Brier score in all scenarios

**Figure 6:**
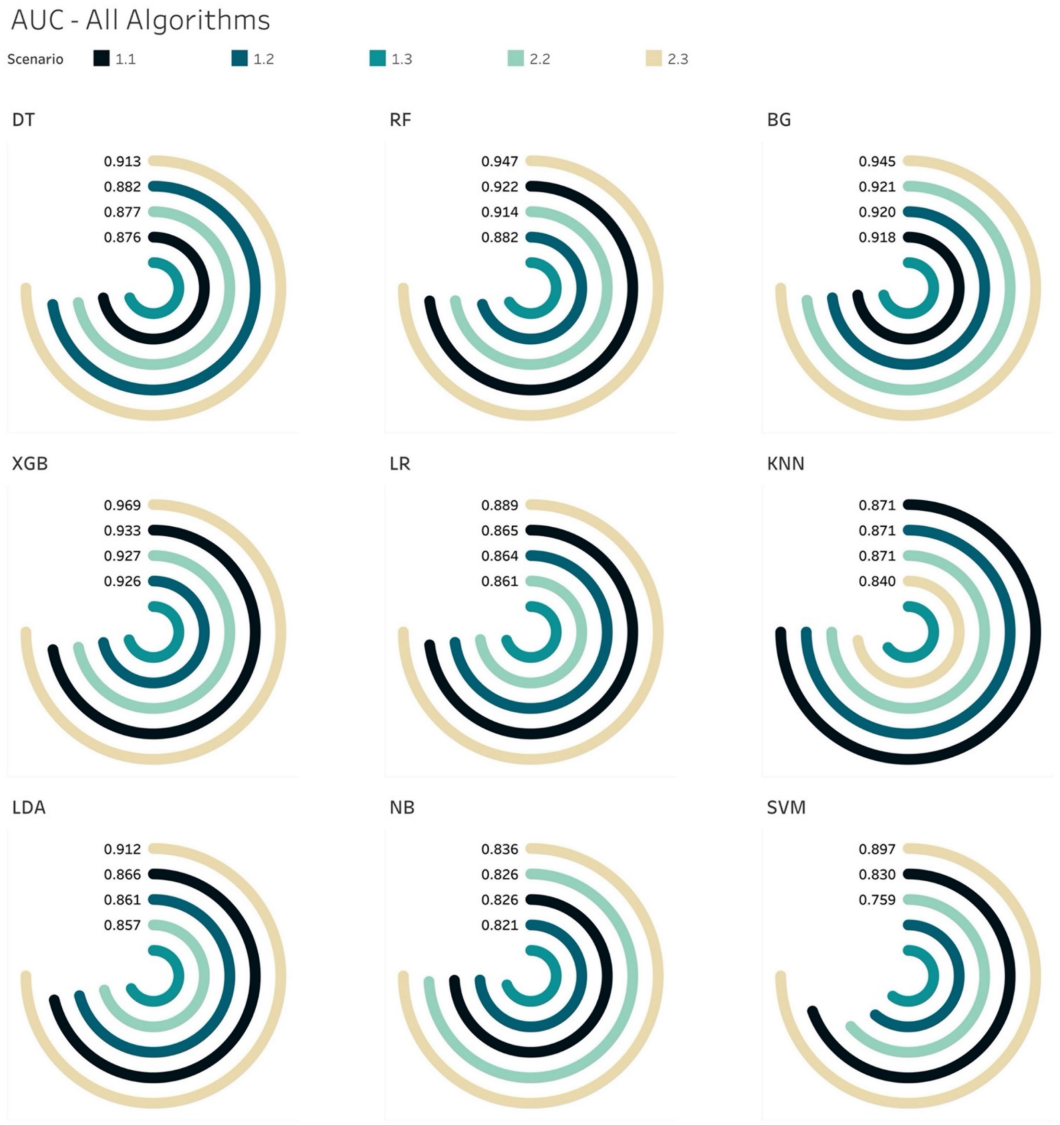
AUCs for all ML models per scenario.

Generally, the tree-based models performed better than non-tree-based ones across all 5 scenarios from the AUCs metric perspective (Figure 6). Among non-tree-based algorithms, only the LDA classifiers achieved AUC scores that were higher than 90% for S2.3.

The AUC results of XGB and BG models yielded the highest scores of 97 and 96%, respectively in S2.3, in which the training and testing sets were both over-sampled. In the under-sampling situations (S1.2 and S2.2), the BG classifier attained AUCs over 90%. Similar results were acquired in the case of XGB, where the AUC for both S1.2 and S2.2 were 92.6 and 92.8%, respectively. Looking deeper, among S1.2 and S2.2, we found out that BG models resulted in similar evaluation scores (Sensitivity, Specificity, Brier score and Log Loss). The same applied to the XGB model (Table 6). This led to an additional finding that the BG and XGB classifiers yield a similar performance when the models endeavour to predict the positive (mortality) outcome when performing a random under-sampling method, regardless of whether the training and the testing distributions of the target class are similar. In other words, the XGB and BG classifiers are not sensitive in predicting the minority class, regardless of the distribution of classes in the target population. Note that DT also yielded a similar pattern (to the XGB and BG classifiers) on both S1.2 and S2.2, however the scores of the evaluation metrics were not as high as those produced by XGB and BG. RF and other non-tree-based classifiers yielded a mixture of Sensitivity, AUC, Brier score and Log Loss values.

**Table 6:** BG and XGB model in 2 scenarios.

Scenario	Model	Sensitivity	Specificity	AUC	Brier score	Log Loss
S1.2	BG	0.890	0.790	0.920	0.053	0.200
S2.2	BG	0.890	0.799	0.921	0.054	0.208
S1.2	XGB	0.884	0.805	0.926	0.050	0.193
S2.2	XGB	0.884	0.803	0.927	0.053	0.205

The analysis thus far gave us another conclusion in relation to all classifiers on various class distributions of the target population. That is that the XGB classifier gave the best performance in terms of discriminating between the 2 classes (survival and mortality) with the highest AUC scores across all scenarios. In the special case of S2.2, BG and XGB had similar AUC scores, where the difference was negligible – 0.61%.

##### 3.1.4.1 Log Loss performance

A similar pattern could be seen in the case of Log Loss metrics across all scenarios per algorithm. The tree-based algorithms generated lower scores on under-sampling sets in comparison to non-tree-based models. However, by examining the Log Loss of all algorithms per scenario, tree-based algorithms attained lower Log Loss scores than the non-tree-based ones under S1.3, S2.2 and S2.3. Moreover, XGB gave the lowest Log Loss score among all algorithms on S2.2, S1.3, and S2.3.

##### 3.1.4.2 Calibration – reliability curves

Tree-based models achieved better calibration in relation to the under-sampling scenarios of S1.2 and S2.2. Non-tree-based classifiers seemed to attain better calibration on the over-sampling scenarios S1.3 and S2.3.

Based on the aforementioned analyses, Autofeat was utilised to remove non-important features for different sampling situations to remodel the selected ML models.

### 3.2 Post dimension reductions

Based on the results prior to utilising Autofeat, RF, XGB, BG and LR were selected to perform dimension reduction. Based on Table 2, Autofeat selected only 33% predictors for the under-sampling scenarios (S1.2 and S2.2), while the over-sampling scenarios (S1.3 and S2.3) considered about 60% of the 67 predictors. The classification metrics of Precision, Recall and F1-score were most balanced in S2.2 and S2.3. This pattern coincided with the scores prior to using Autofeat. In other scenarios, those scores appeared to fluctuate in the range 0.44 to 0.96.

Table 7 provides the details of monitoring the changes in the AUC, Brier score and Log Loss. The AUC percentages when using XGB were greater than 0.9 on all samples. Noticeably, when testing on S2.3, the AUC scores for the XGB classifier remained unchanged, while Brier score and Log Loss reduced slightly when compared to their values pre-dimension reduction. For BG, the AUC percentage also was the same in S2.2, while both Brier score and Log Loss were smaller after considering much fewer number of predictors.

**Table 7:** Changes in metrics post dimension reduction.

Scenario	Model	Precision	Recall	F1-score	Sensitivity	Specificity	AUC	Brier score	Log Loss
S1.1	RF	0.846↓	0.743↓	0.774↓	0.473↓	0.968↓	0.911↓	0.024**↑**	0.117**↑**
S1.2	BG	0.880↓	0.799↓	0.821↓	0.850↓	0.789↓	0.906↓	0.054**↑**	0.208**↑**
S1.2	XGB	0.879↓	0.799↓	0.821↓	0.850↓	0.789↓	0.909↓	0.054**↑**	0.206**↑**
S2.2	BG	0.830↓	0.829↓	0.829↓	0.850↓	0.808**↑**	0.908↓	0.054**↑**	0.212**↑**
S2.2	XGB	0.820↓	0.819↓	0.818↓	0.850↓	0.787↓	0.908↓	0.056↓	0.216↓
S2.3	XGB	0.899	0.896	0.896**↑**	0.855**↑**	0.937↓	0.967↓	0.017	0.094**↑**
S1.3	XGB	0.880**↑**	0.883	0.881**↑**	0.602**↑**	0.937↓	0.904↓	0.017	0.095**↑**
S1.3	LR	0.850↓	0.629**↑**	0.677**↑**	0.868↓	0.583**↑**	0.823↓	0.067↓	0.263↓

#### 3.2.1 Calibration or reliability curve

**Figure 7:**
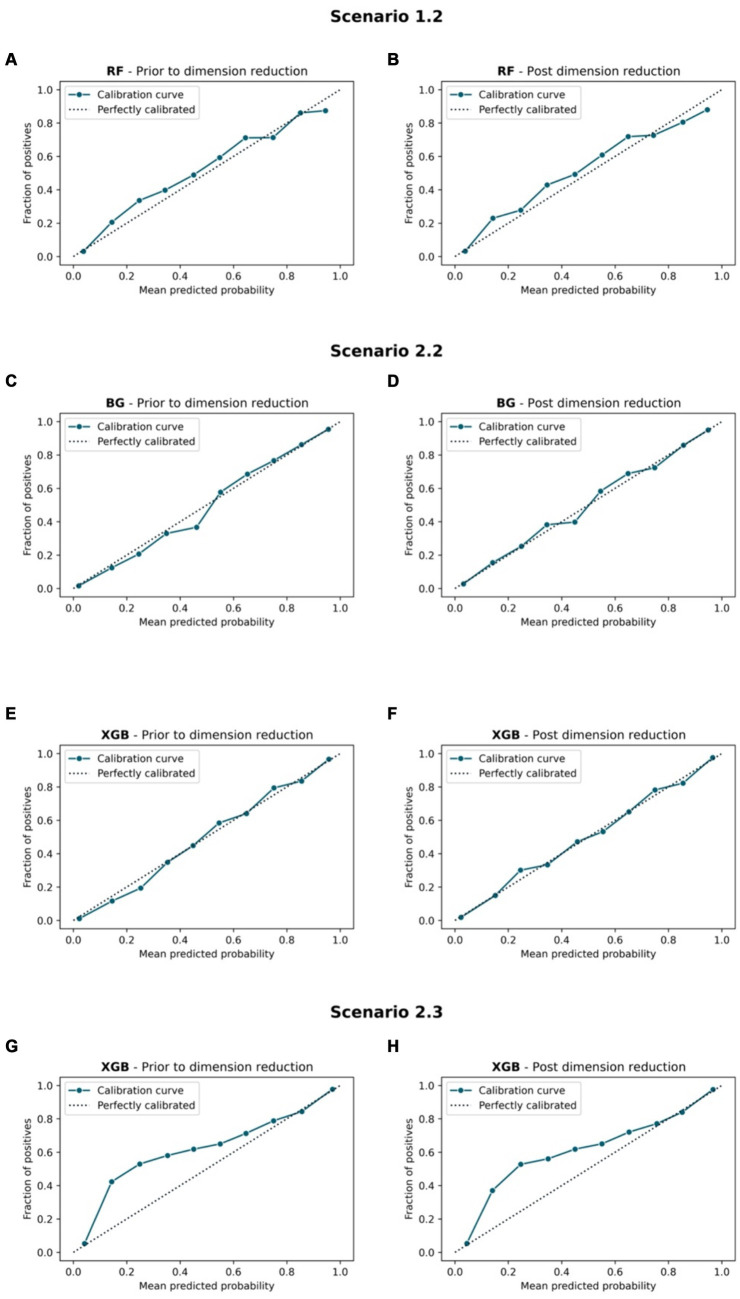
Calibration curve. Scenario 1.1, **(A)** RF model prior to dimension reduction and **(B)** RF model post dimension reduction. Scenario 2.2, **(C)** BG model prior to dimension reduction, **(D)** BG model post dimension reduction, **(E)** XGB model prior to dimension reduction, and **(F)** XGB model post dimension reduction. Scenario 2.3, **(G)** XGB model prior to dimension reduction and **(H)** XGB model post dimension reduction.

**Figure 8:**
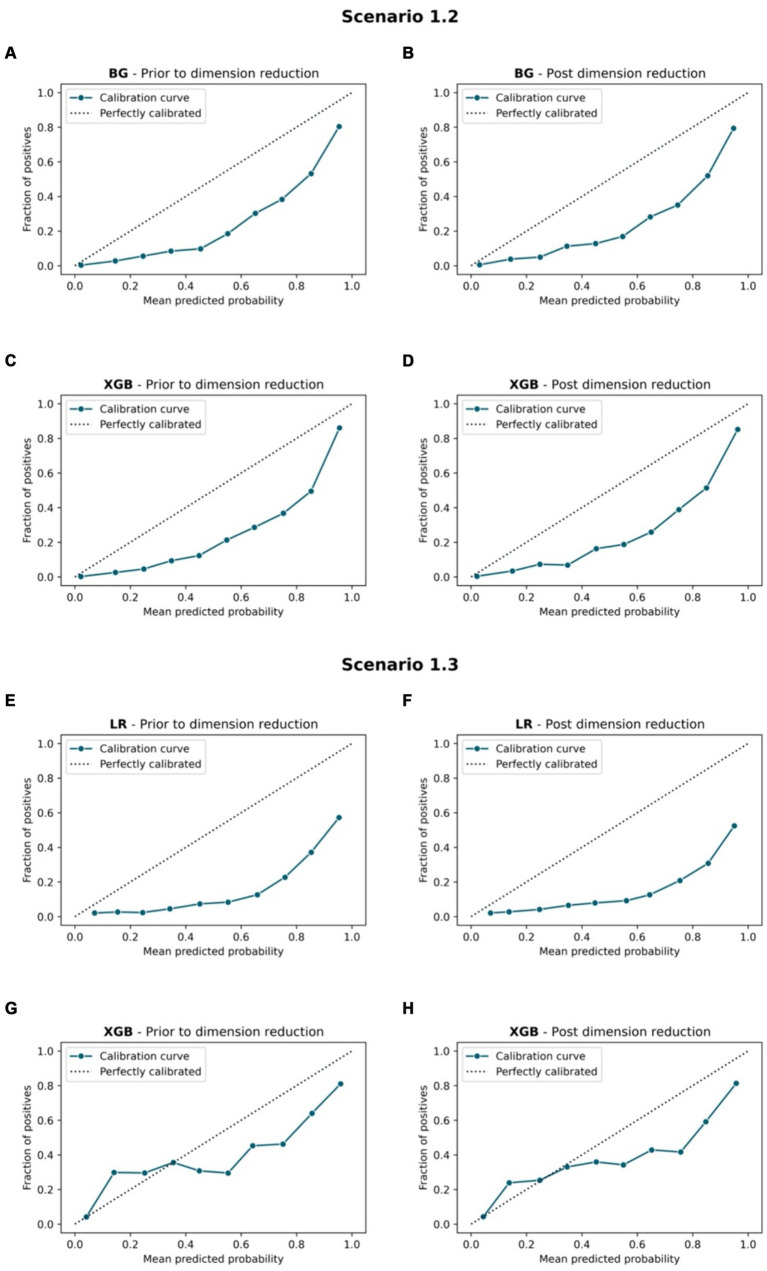
Calibration curve. Scenario 1.2, **(A)** BG model prior to dimension reduction, **(B)** BG model post dimension reduction, **(C)** XGB model prior to dimension reduction and **(D)** XGB model post dimension reduction. Scenario 1.3, **(E)** LR model prior to dimension reduction, **(F)** LR model post dimension reduction, **(G)** XGB model prior to dimension reduction, and **(H)** XGB model post dimension reduction.

Figures 7, 8 compare the calibration prior to and post dimension reduction of the best models in each scenario. Figure 7 shows that the XGB and BG classifiers of S2.2 resulted in the most aligned calibration curve to the diagonal line, while the reliability curve for BG showed that the model over-predicted at low probabilities and under-estimated at high probabilities. In fact, the calibration curve of XGB classifier in S2.2, post Autofeat, was slightly more aligned to the calibration curve than prior to using it. This entails that by using fewer features, the predicted probability of the XGB classifier may be more accurate than using larger number of predictors.

The MCC showed that post reducing dimensions, were slightly smaller across all scenarios and all ML models (Table 4). Similar situations can be found with the AUC 95% CI metrics. This confirms the fact that removing features using Autofeat did not change the prediction accuracy of the selected ML models.

The list of predictors that were used for the 3 main different situations, including imbalanced class, under-sampling and over-sampling techniques are attached as Supplementary material S3.

## 4 Discussion

The purpose of this study was to use 9 ML algorithms to identify the best ML model that can discriminate between the classes and provide relatively high accurate predicted probability (death = positive, survival = negative). While this study considered a number of evaluation metrics, such as Precision, Recall, F1-score, Sensitivity, Specificity, AUC, ROC curve, and Calibration curve, Brier score and Log-loss values were also considered. Since our study would select models with high Sensitivity among those that have similar AUC score or Brier scores, this study presented the following findings:

- The key finding of our study, utilising 9 ML methods across 5 different scenarios, is the successful development of well-performing ML models using a significantly reduced dataset size for the prediction task. Specifically, the original size of the dataset was 872,388 (13,218 observations and 67 predictors for the training set of S1.1). Following dimension reduction, ML models required only about 11% of the total data points to perform prediction task effectively in S2.2, with a training size of 4,204 observations and 22 features. This indicates that the original dataset might have contained redundant information. By using Autofeat, which is independent of the behaviour of ML models, clinically important features were retained.
- The study used the MIMIC-III data of ICU patients 24 h after hospital admission in order to construct and analyse patient mortality prediction models. Related analysis results revealed that, in general, tree-based classifiers outperformed non-tree-based ones in terms of model prediction accuracy.
- Zhu et al. (7) established the fact that the XGB model performed the best among all 7 models since it tested on the unseen data and produced an AUC of 0.821. In our study, we also concluded that the XGB classifier yielded the highest values for the majority of the evaluation metrics except for the imbalanced class situation in S1.1. Our study also showed that the AUCs obtained from the different test sets were all above 0.9.
- Calibrated XGB and BG models are most aligned to the diagonal line on the under-sampling sets in comparison to other sampling sets, but have poorer Brier and Log Loss Scores. This suggests that if using XGB and BG algorithms, the predicted probabilities are likely to be better aligned with the actual probabilities, but these models might lack some Precision in terms of distinguishing between different classes.
- The number of feature groups remained the same as prior to using Autofeat. Moreover, the features within the demographic characteristics and disease severity groups were retained in all scenarios in the post dimension reduction models. These 2 groups, considered to be important in clinical diagnosis and prognosis, were effectively preserved by Autofeat across different sampling scenarios. This finding is supported by several studies that have utilised these 2 groups of features in clinically predicting hospital mortality among mechanically ventilated patients in ICUs, such as Jones et al. (22), Choudhry et al. (23), Souza-Dantas et al. (24) and Gadre et al. (25).
- The results of the 3 tree-based and 1 non-tree-based models showed that the reduced models can achieve similar accuracy metrics as those using a much larger number of predictors. In fact, the AUCs remain as high as 0.90 across 4 scenarios (S1.2, S1.3, S2.2, and S2.3) for the tree-based models. The full model used 67 predictors, while the reduced model for the imbalanced case used 29 predictors, 22 for the under-sampling scenarios and 40 for the over-sampling scenarios. These results demonstrated the rapid development of ML algorithms and that they can provide significant assistance to physicians when making clinical decisions. This finding coincided with those by Chiu et al. (26).
- Non-tree-based calibrated models resulted in mixed performance on both prediction and prediction reliability under different scenarios. The LR model yielded a relatively high Sensitivity and AUCs scores (> 0.8) prior to and post dimension reduction in S1.3.
- In the case of under-sampling scenarios (S1.2 and S2.2), the Recall and F1 scores for the mortality group were higher than for the survival group, while in the case of over-sampling scenarios (S2.3 and S1.3), the Recall and F1 scores for the survival group were higher than. These scores were replicated in all models, except SVM. This finding entails that regardless of different distributions of a target population, if the purpose is to achieve high Recall scores for the minority, an under-sampling method should be preferred.

Note that we are aware that LR does not usually require any extra post-training calibration as the probabilities it produces are already well-calibrated. Conversely, RF classifiers seldom return values close to 0 or 1 because they generate average responses from multiple inner models – the only way of achieving boundary values (0 or 1) in this case is if all the models return values close to 0 or 1 – a rare event from the probabilistic standpoint. Therefore, assessing a calibrated RF classifier might not be helpful. In this study, we assessed and evaluated all calibrated models to compare the performance. The results of this study became the foundation of our next investigation, which is to focus on the usage of the compact ML models to predict the outcome of mechanically ventilated patients of 7 days and 28 days.

## 5 Limitations

This study built prediction models solely focused on mechanically ventilated patients. Therefore, the models are only applicable to ICU patients who require mechanical ventilation. This issue of lack of generalisability is a common problem when constructing prediction models by using ML methods and dynamic EHR data (26).

Another issue concerns the single-central nature of the MIMIC-III database. Since the data were collected in Boston, MA, United States, the medical practices for ICU patients, healthcare policies, and demographic characteristics might be different from other locations. Future studies focusing on validating the models using data of mechanically ventilated patients in other countries can result in a more robust utilisation of those models and facilitate their application in broader healthcare settings. Additionally, the ICD-9 codes used for identifying patients’ medical history were assigned post-hospital discharge based on a review of clinical notes. Consequently, it might not be valid to assume that a patient was coded with a pre-existing condition upon admission, or the condition developed during their ICU stay. This ambiguity introduces a potential confounding factor in mortality prediction, particularly when considering laboratory result variables and vital sign variables. The inclusion of pre-existing conditions in the models may distort the association between these variables and mortality risk, as chronic conditions may influence the predictive power of acute physiological markers.

Our study did not investigate cases where the prevalence of the minority group was less than 2%. This ratio might worsen the performance of the models.

## 6 Conclusion

In this study, in addition to building the best ML models to predict the mortality outcomes of mechanically ventilated patients, we automated the process of feature engineering and yield models that used only 1/3 of the predictors, while maintaining similar accuracy as that achieved by models that used 67 predictors. This finding acts as the evidence of possible building and deploying compact ML models in reality with high accuracy, and less cost to monitor input data and computational resources. However, the accuracy probability of those models was either over- or under-estimated the mortality outcome. Therefore, a follow-up studies are required to improve the probability accuracy.

One benefit to mechanically ventilated patients and their support networks, when hospitals implement such ML applications, is the facilitation of collaborative decision-making between patients’ relatives or patients themselves and clinicians. In critical moments, these individuals can use ML applications to make informed decisions, potentially preventing unnecessary admissions and mitigating healthcare expenses for the patients.

Holistically, our research findings demonstrated that ML applications in healthcare can enhance decision support systems and operate in a timely manner. As a result, the clinical operations can be optimised in terms of finance, resources and potentially, human lives. Van Wyk et al.’s cost–benefit analysis (27) focusing on automated data extraction and the utilisation of ML concluded that powerful ML algorithms, along with automated data acquisition, can offer immense societal benefits. Despite such benefits, challenges associated with ML applications, such as bias and privacy concerns, cannot be overlooked.

## Data availability statement

The datasets presented in this study can be found in online repositories. The names of the repository/repositories and accession number(s) can be found at: https://mimic.physionet.org.

## Ethics statement

Ethical review and approval was not required for the study on human participants in accordance with the local legislation and institutional requirements. Written informed consent from the patients/ participants was not required to participate in this study in accordance with the national legislation and the institutional requirements.

## Author contributions

QN: Conceptualization, Formal analysis, Methodology, Project administration, Supervision, Writing – original draft, Writing – review & editing. MT: Data curation, Formal analysis, Investigation, Software, Visualization, Writing – review & editing, Validation. VP: Data curation, Formal analysis, Investigation, Software, Validation, Visualization, Writing – review & editing. AL: Data curation, Formal analysis, Investigation, Project administration, Software, Validation, Writing – review & editing. GN: Investigation, Methodology, Resources, Writing – review & editing, Conceptualization.

## Glossary

AUC: Area Under the Curve
BG: Bagging
DFS: Deep Feature Synthesis
DT: Decision Tree
ICU: Intensive Care Unit
KNN: K-Nearest Neighbour
LDA: Linear Discriminant Analysis
LR: Logistic Regression
MCC: Matthew Correlation Coefficients
ML: Machine Learning
NB: Naïve Bayes
RF: Random Forest
ROC: Receiver Operating Characteristic
S1.1: Scenario 1.1
S1.2: Scenario 1.2
S1.3: Scenario 1.3
S2.2: Scenario 2.2
S2.3: Scenario 2.3
SMOTe: Synthetic Minority Over-sampling Technique
SVM: Support Vector Machine
XGB: eXtreme Gradient Boosting
